# Topical Ocular TRPV1 Antagonist SAF312 (Libvatrep) for Postoperative Pain After Photorefractive Keratectomy

**DOI:** 10.1167/tvst.12.3.7

**Published:** 2023-03-14

**Authors:** Vance Thompson, Majid Moshirfar, Thomas Clinch, Stephen Scoper, Steven H. Linn, Avery McIntosh, Yifang Li, Matt Eaton, Michael Ferriere, Kalliopi Stasi

**Affiliations:** 1 Vance Thompson Vision, Sioux Falls, SD, USA; 2 University of South Dakota, Sanford School of Medicine, Sioux Falls, SD, USA; 3 Hoopes, Durrie, Rivera Research, Hoopes Vision, Draper, UT, USA; 4 Eye Doctors of Washington, Washington, DC, USA; 5 Virginia Eye Consultants, Norfolk, VA, USA; 6 Novartis Pharmaceuticals Corp., East Hanover, NJ, USA; 7 Novartis Institute of Biomedical Research, Cambridge, MA, USA

**Keywords:** libvatrep, SAF312, TRPV1, PRK, ocular surface pain, cornea, VAS

## Abstract

**Purpose:**

Evaluation of safety and efficacy of topical ocular SAF312 (Libvatrep) in post–photorefractive keratectomy (PRK) pain.

**Methods:**

In this placebo (vehicle)–controlled, participant- and investigator-masked study, 40 participants were randomized (1:1) to two treatment sequences in a bilateral PRK crossover design (SAF312 2.5% followed by vehicle [or vice versa], one eye drop, four times daily for 72 hours after PRK). Primary endpoints were visual analog scale (VAS) pain scores at 6 hours after first drop of study drug and average VAS scores over 0 to 12 hours postoperatively. Secondary endpoints included postoperative oral rescue medication (ORM) use and adverse events (AEs).

**Results:**

All 40 participants completed the study. Both primary endpoints were met; mean difference in VAS pain scores between SAF312- and vehicle-treated eyes was −11.13 (*P* = 0.005, −25%) at 6 hours postoperatively and −8.56 (*P* = 0.017, −22%) over 0 to 12 hours. Mean VAS pain scores with SAF312 were consistently lower than with vehicle from 1 hour postoperatively up to 30 hours (*P* ≤ 0.10 observed in 8/11 time points). Less ORM was taken with SAF312 up to 0 to 72 hours postoperatively, with a trend of fewer participants taking ORM at 0 to 24 hours postoperatively with SAF312 versus vehicle. No serious AEs were reported. All ocular AEs were mild and transient, and none were drug related. SAF312-treated eyes showed no delay in wound healing and had a lower grade 4 conjunctival hyperemia 24 hours postoperatively versus vehicle-treated eyes.

**Conclusions:**

SAF312 was well tolerated and effective in reducing ocular pain post-PRK.

**Translational Relevance:**

Topical SAF312 presents a new therapeutic option for patients undergoing PRK.

## Introduction

The cornea is one of the most densely innervated structures in the human body, with a dense subepithelial plexus in the anterior third of the stroma and sensory afferent neurons that send painful stimuli to the central nervous system (CNS).^1^^,^^2^ Photorefractive keratectomy (PRK) is a corneal refractive surgery performed to correct visual refractive errors and reduce or eliminate dependency on glasses or contact lenses. Although PRK is safe and effective, it is frequently associated with postoperative pain, prolonged visual recovery, and delayed healing time.^3^^,^^4^ Pharmacologic pain management options after PRK include various combinations of on-label topical nonsteroidal anti-inflammatory drugs (NSAIDs), topical corticosteroids, and off-label diluted local analgesics, including topical morphine,^5^ together with oral opioids and analgesics (painkillers).^4^^,^^6^^–^^8^ The use of topical ocular agents is preferred due to lower systemic side effects.^4^ Currently, only the topical ocular NSAIDs diclofenac (0.1%) and ketorolac (0.4% and 0.5%) have been approved by the US Food and Drug Administration (FDA) for pain management following PRK.^3^^,^^9^ Although effective in reducing pain, topical NSAIDs can result in complications such as delayed corneal reepithelialization, corneal toxicity, corneal melting, and corneal scarring.^10^^–^^16^ Diclofenac showed a direct anesthetic effect on pain nerve activity in cats^17^ but did not show clinically significant cornea anesthetic effect in healthy volunteers with Cochet–Bonnet esthesiometry.^18^ Diclofenac 0.1% was recalled from the US market in 1999 due to reports of corneal perforations.^19^ Common practice postoperative treatments, including oral opioids, are associated with several systemic side effects, and prolonged use increases the risk of addiction.^4^^,^^20^ Novel pain management strategies with fewer side effects are needed to alleviate postoperative pain following PRK.

Transient receptor potential cation channel subfamily V member 1 (TRPV1) is a key nociceptor in corneal tissues that has a dual function in pain sensing, transmission, and regulation, as well as mediating innate inflammatory responses.^21^^,^^22^ TRPV1 channel inhibitors directly block the pain-generating receptor and have been shown to be effective in reducing inflammatory, traumatic, nociceptive, and neuropathic pain in both humans and animals.^23^^–^^25^ SAF312 is a potent, highly selective noncompetitive TRPV1 antagonist that is currently under investigation for use as topical ocular analgesic eye drops.^26^ A first-in-human (FIH) dose-ascending phase 1 clinical trial in healthy volunteers showed that topical ocular SAF312 had a well-tolerated ocular and systemic safety profile, with no anesthetic effect up to the maximum feasible concentration (2.5%) at the supratherapeutic dose of eight times daily for 7 days.^18^ The objective of this phase 2 proof-of-concept (PoC) study was to evaluate the safety and efficacy of topical ocular SAF312 2.5% on pain control in the immediate postoperative period following PRK.

## Methods

### Study Design and Treatment

This phase 2, participant- and investigator-masked, randomized, placebo (vehicle)–controlled study (NCT02961062) was conducted across four centers in the United States from 2016 to 2018. The study was approved by the institutional review boards of all clinical sites and adhered to the tenets of the Declaration of Helsinki. Written informed consent was obtained from all participants.

**Figure 1.**
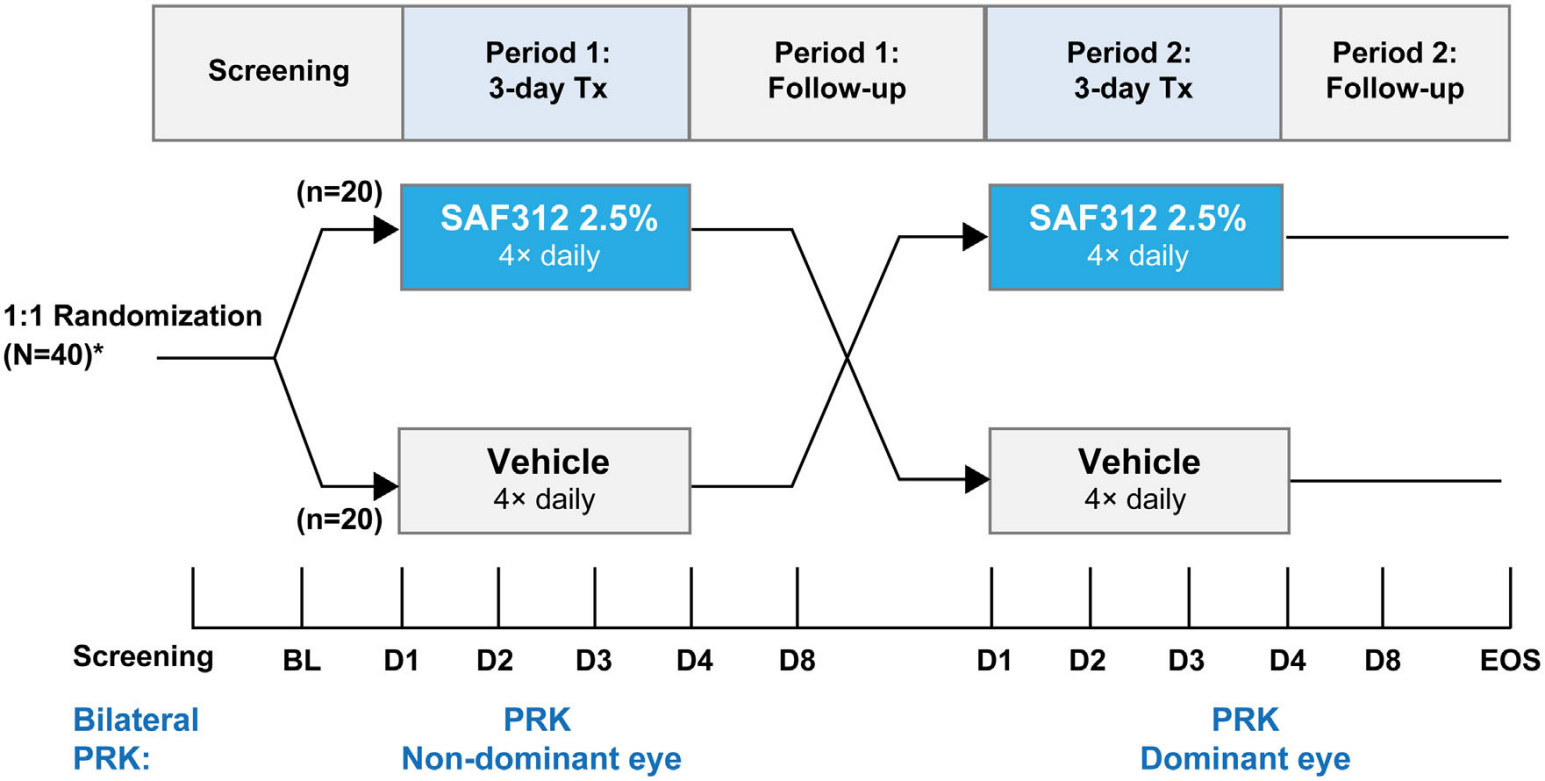
Study design: phase 2, randomized, masked, crossover trial. *As a result of the crossover design, each participant served as their own control (one eye treated with SAF312 and the other eye treated with vehicle). PRK surgery was first performed in the participant's nondominant eye, as determined at screening and agreed upon by the participant and the investigator. Site staff administered the first study dose inside the operating room at the conclusion of the surgery, and subsequent doses were self-administered. The participant returned for follow-up visits on days 2, 3, 4, and 8 of period 1 after surgery in the first eye, with optional daily visits to monitor the participant until wound healing was complete. The second eye surgery (dominant eye) was performed in period 2 (after the resolution of the epithelial defect in the first eye). After the second surgery (period 2), participants received opposing study drug treatment for 72 hours at same dosing schedules and follow-up visits as in period 1. BL, baseline; D, day; *N*, total number of participants; *n*, number of participants; Tx, treatment.

The study used a bilateral crossover design^27^ with 40 participants randomized, via Interactive Response Technology at baseline, in a 1:1 ratio to two treatment sequences: SAF312 in period 1 following PRK in the nondominant eye, followed by vehicle in period 2 after PRK in the fellow eye or vice versa with treatment ordering (Fig. 1). Both SAF312 and vehicle were administered as a single eye drop four times daily (every 6 hours) in one eye from immediately postoperatively inside the operating room (time 0) up to the last dose at 72 hours. All participants received the standard-of-care postoperative treatment of bandage contact lens (Air Optix Night and Day Aqua, Alcon, Texas, USA or equivalent), followed by topical ocular antibiotics (moxifloxacin [or equivalent], one eye drop four times daily) after the first dose of study drops and continued for 4 to 7 days; prednisolone acetate ophthalmic, one eye drop four times daily, administered for 1 week after PRK and followed by taper per local procedures; artificial tears (preservative free) as needed and per local practice and per patient; and oral rescue medication (ORM, acetaminophen 300 mg plus codeine 30 mg) as needed up to a total of 10 tablets/d or 1 to 2 tablets every 4 hours. No other pain medication was allowed 24 hours prior to and up to 4 days postoperatively. NSAID eye drops were not allowed at any time.

### Eligibility Criteria

Male and female participants aged 18 to 75 years who were eligible for bilateral PRK (1) had a normal eye exam except for refractive error at baseline, and (2) the planned myopia correction did not exceed −4.00 diopters (sphere) and 3.00 diopters of astigmatism, with spherical equivalent not higher than −4.50, confirmed by manifest refraction at baseline.

The complete exclusion criteria are provided in Supplementary Appendix 1.

### Objective, Endpoints, and Assessments

The primary objective was to evaluate pain control in the immediate post-PRK period. The coprimary endpoints were (1) visual analog scale (VAS) pain assessment at 6 hours postoperatively and (2) average ocular pain VAS assessments from the first postoperative assessment up to the 12-hour predose assessment. Secondary endpoints included (1) incidence and amount of ORM needed from 6 hours to 3 days postoperatively after each PRK surgery; (2) adverse events (AEs) and serious AEs (SAEs); (3) size of epithelial defect by slit-lamp exam, visual acuity (VA), blink rate, and tear production; (4) all VAS measurements during the first 3 days after surgery; and (5) plasma concentration and pharmacokinetic (PK) parameters of SAF312.

To account for the potential influence of the rescue medication on the primary endpoints, three different approaches of analyzing the VAS pain scores and calculating the average (0–12 hours) were planned in advance and evaluated after data collection. The three different approaches included any recorded VAS pain score at time points within 4 hours after the use of rescue medication, which was imputed by the record taken at the time of rescue medication use. This method was applied as the primary sensitivity analysis, as per the FDA guidance in 2014.^28^ Any recorded VAS pain score at time points within 4 hours after the use of rescue oral analgesics was considered missing (not imputed), and all the recorded VAS pain scores were used in both primary efficacy models.

The exploratory endpoint included is the Ocular Pain Assessment Survey (OPAS) outcome. OPAS was designed and validated to measure ocular pain and quality of life in patients with corneal and ocular surface pain at initial and follow-up visits in the clinic over a 6-month period.^29^ The etiology of pain included infectious and noninfectious keratitis, corneal ulcers, dry eye disease, ocular graft-versus-host disease, allergic conjunctivitis, keratoconus, and refractive surgery.^29^ OPAS has been tested only in a minority of patients after refractive surgery but not immediately after PRK. Hence, it was considered only a tertiary exploratory endpoint in this study. OPAS was not powered for detection of any difference, and no statistical analysis was planned or performed. Patients were asked to fill out the OPAS questionnaire 24 hours, 72 hours, and 8 days after PRK surgery.

VAS pain assessments were captured digitally in real time with a handheld (electronic patient-reported outcome [ePRO]) device at the protocol-specified time points. VAS uses a numeric assessment of pain between 0 and 100, with 0 representing no pain and 100 representing the worst imaginable pain. Safety assessments consisted of AEs and SAEs, with their severity and relationship to the study drug. Ocular hyperemia was assessed using the McMonnies redness scale (0–5 photographic scale, with higher scores indicating a greater degree of conjunctival redness) in four regions of the bulbar conjunctiva (superior, inferior, temporal, nasal). Each eye and each region were graded by severity (0–5).^30^

The details of other assessments performed in the study are provided in Supplementary Appendix 2.

### Statistical Analyses

This study was powered based on the difference in mean 6-hour VAS score because there were insufficient data to calculate an exact sample size for the second coprimary endpoint of average VAS from 0 to 12 hours. Further, with a prespecified false-positive rate (α) for all tests of 0.10, a *P* value ≤0.10 was considered statistically significant. The statistical power of the study was expected to be higher than a traditional two-group design due to the bilateral crossover design,^31^ which accounts for the potentially high correlation of efficacy results between both eyes from the same participant; within-participant variability (in pain perception) is expected to be much smaller than between-participant variability. Of the 40 participants randomized in a 1:1 ratio between two treatment sequences, 10 patients were not included in the primary endpoint analysis due to nonevaluable data (after technical failure of the initial ePRO device), resulting in an ∼83% power to reject the null hypothesis of equality of mean VAS at the 0.1 level of significance (two-sided) in a unilateral parallel design, assuming a treatment difference of 20 mm and standard deviation (SD) of 30 mm at 6 hours postoperatively.^32^

VAS pain assessments were analyzed 6 hours postoperatively using a linear mixed model for repeated measures, which accounted for the crossover nature of the trial. VAS pain assessment up to 12 hours postoperatively was assessed with a mixed-effect model accounting for the crossover effect for average (0–12 hours) VAS. The difference in least squares mean VAS scores between SAF312-treated eyes and vehicle-treated eyes, the 90% confidence interval, and *P* value testing treatment difference at a given time point were obtained from the model at each time point.

To assess the secondary endpoint of oral rescue medication use, McNemar's test was used to analyze the difference between the number of participants who did not take oral analgesics between SAF312 and vehicle treatment during the intervals of interest (0–6, 0–12, 0–24, 0–48, and 0–72 hours postoperatively); the associated *P* values were reported. The amount (mg/kg body weight, and number of pills) of oral analgesics taken in each period by each participant was analyzed by the Wilcoxon signed-rank test; the associated *P* values were reported. Patient demographic and baseline characteristics, safety, and PK analyses were summarized descriptively.

## Results

### Participant Disposition

All 40 randomized participants completed the study and were included in the safety analysis set. The first 10 randomized participants were excluded from the primary endpoint analysis (5 participants each from both treatment sequences) due to an ePRO device failure; the primary and secondary endpoints were successfully collected in the next 30 participants using a completely different ePRO device. All 40 randomized participants were included in the secondary endpoint and safety analysis sets (Supplementary Table S1).

### Participant Baseline Demographics and Characteristics

The demographic characteristics were well balanced between the participants of both treatment sequences. The mean age of the participants was 34 years (range, 20–56), 53% were male, and the majority were Caucasian (88%) (Table 1).

**Table 1.** Participant Demographics

Characteristic	Vehicle/SAF312 2.5% (*N* = 20)	SAF312 2.5%/Vehicle (*N* = 20)	Total (*N* = 40)
Gender, *n* (%)			
Female	9 (45)	10 (50)	19 (48)
Male	11 (55)	10 (50)	21 (53)
Age, y			
Mean (SD)	34.4 (10.77)	33.7 (8.94)	34.0 (9.78)
Race, *n* (%)			
Caucasian	18 (90)	17 (85)	35 (88)
Asian	2 (10)	1 (5)	3 (8)
Black or African American	0 (0)	1 (5)	1 (3)
Multiple	0 (0)	1 (5)	1 (3)
BMI, kg/m^2^			
Mean (SD)	28.9 (3.98)	26.2 (3.73)	27.5 (4.04)

Safety analysis set.

BMI, body mass index; *N*, total number of participants; *n*, number of participants.

### Primary Outcome: VAS Pain Assessment

**Figure 2.**
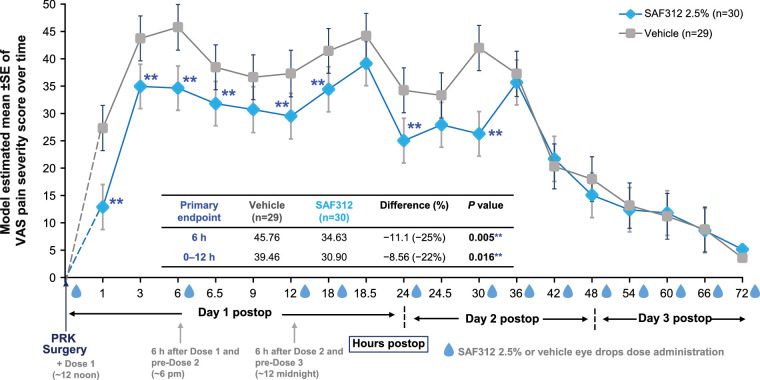
SAF312 2.5% four times daily lowered VAS pain scores versus vehicle (placebo) consistently starting at 1 hour after dosing and during the first 30 hours after PRK. Both primary endpoints (VAS pain at 6 hours and over 0–12 hours) were met. VAS data from the first 10 participants were not evaluable due to failure of the ePRO device. Plotted means are least squares means from the primary efficacy analysis of VAS using a mixed-model repeated measures approach. Any recorded VAS pain score at time points within 4 hours after ORM use was replaced by the VAS record taken immediately before ORM use. **Statistically significant at α = 0.10. *n*, number of participants; post-op, postoperative.

Both primary efficacy endpoints met the prespecified statistical significance threshold. The mean difference in VAS pain severity scores between SAF312- and vehicle-treated eyes was −11.13 (*P* = 0.005, −25%) at 6 hours postoperatively and −8.56 (*P* = 0.017, −22%) averaged over 0 to 12 hours postoperatively, respectively (Fig. 2). From the first time point checked at 1 hour after PRK and in all scheduled time points up to and including 36 hours after surgery, mean VAS pain severity scores were consistently lower in eyes treated with SAF312 compared to vehicle, with statistical significance (*P* ≤ 0.10) observed in 8 of 11 time points from 1 to 30 hours postoperatively. From 36 to 72 hours after PRK, the differences in VAS scores between SAF312 and vehicle were nominal (insignificant) with mild pain or less in both treatment groups (Fig. 2 and Supplementary Table S2). The study participants recorded the exact time and amount of any ORM that they took after the procedure. Overall, 20% of evaluable VAS scores were recorded within 4 hours after a recorded ORM use. In this case, to decouple the effect of ORM on VAS primary efficacy endpoints, prespecified analysis was performed, with VAS reading taken within 4 hours of any ORM replaced with the VAS value recorded at the time the ORM was taken (Fig. 2).

### ORM Use

The mean number of ORM (number of acetaminophen/codeine tablets) taken during SAF312 treatment was fewer at every time interval compared to vehicle, with statistically significant (*P* ≤ 0.10) differences observed during the intervals of 0 to 6, 0 to 24, 0 to 48, and 0 to 72 hours postoperatively (Table 2). The amount of ORM taken per body weight (mg/kg) was also lower during SAF312 compared to vehicle treatment at all time intervals (Supplementary Table S3).

**Table 2.** Amount of Rescue Medication Used for SAF312- and Vehicle-Treated Eyes at All Postoperative Intervals

Characteristic	SAF312 2.5% (*N* = 40)	Vehicle (*N* = 40)	% Difference	*P* Value
0–6 hours postoperatively				
Mean (SD)	0.75 (1.032)	1.00 (1.132)	−25	0.10^a^
Range	0.0–4.0	0.0–4.0		
0–12 hours postoperatively				
Mean (SD)	1.40 (1.780)	1.65 (1.847)	−15	0.26
Range	0.0–6.0	0.0–6.0		
0–24 hours postoperatively				
Mean (SD)	2.35 (2.751)	2.80 (3.006)	−16	0.05^a^
Range	0.0–10.0	0.0–12.0		
0–48 hours postoperatively				
Mean (SD)	4.05 (4.466)	4.68 (5.225)	−13	0.05^a^
Range	0.0–16.0	0.0–20.0		
0–72 hours postoperatively				
Mean (SD)	4.33 (4.896)	5.05 (5.574)	−14	0.07^a^
Range	0.0–21.0	0.0–22.0		

Secondary analysis set.

% Difference is calculated by ORM use of (SAF312 – vehicle)/vehicle. *P* value compares the mean between both treatment groups, obtained from the Wilcoxon signed-rank test.

a Statistical significance with *P* ≤ 0.10 (per prespecified primary endpoint α level).

**Figure 3.**
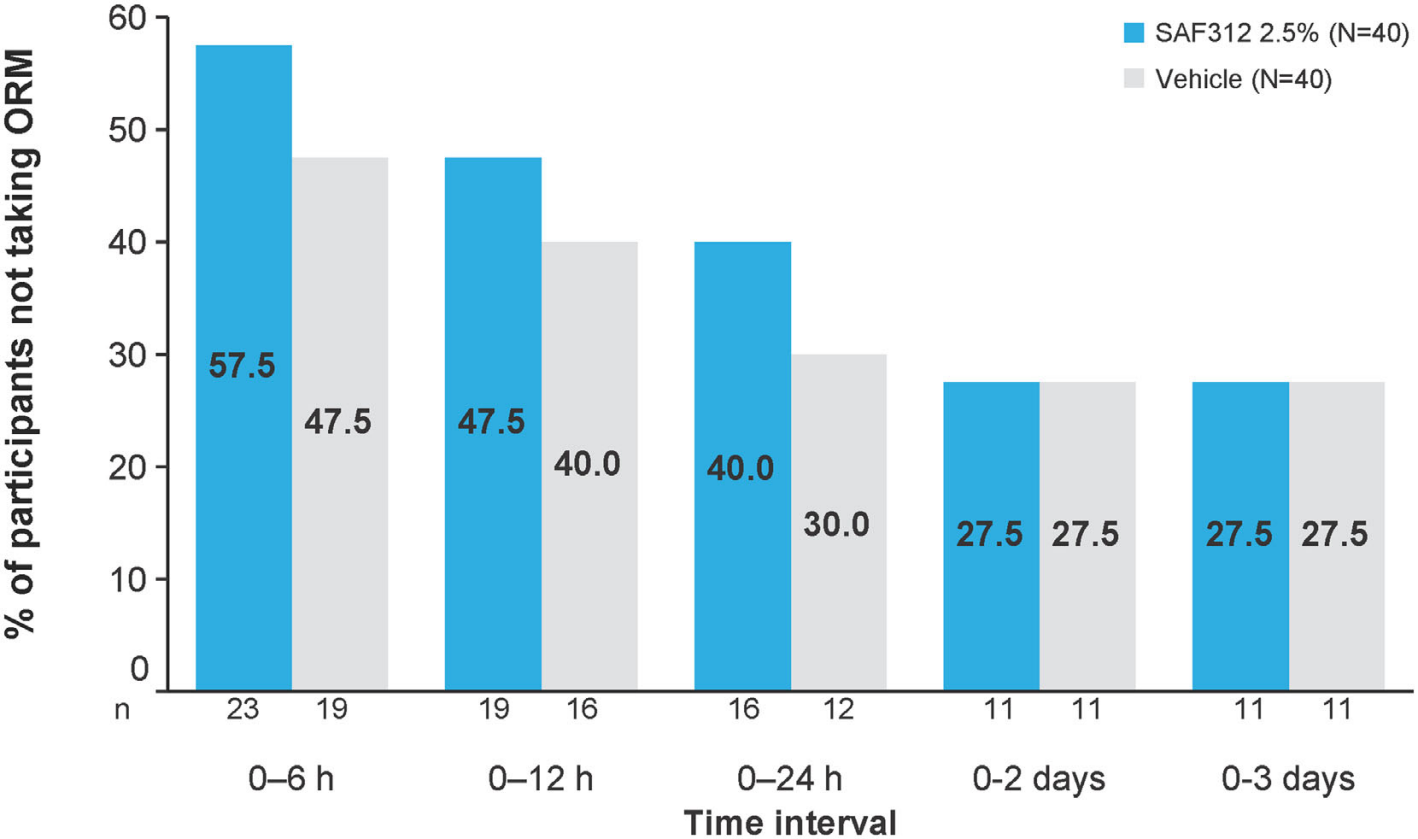
More participants did not use ORM (pain was controlled with eye drops alone) during the first 24 hours postoperatively in SAF312-treated eyes than in the vehicle-treated eyes. Secondary analysis set. For both treatment sequence columns, n represents the number of participants who did not use ORM in any treatment group sequence. *P* value compares the mean of both treatment groups, obtained from McNemar's test; *P* values for all time points were greater than 0.1. *N*, total number of participants; *n*, number of participants.

The number of participants who did not use ORM was higher in SAF312-treated eyes compared with vehicle-treated eyes during the 0- to 6-hour, 0- to 12-hour, and 0- to 24-hour postoperative period. The same number of participants had ORM in SAF312 versus vehicle treatment groups from 0 to 2 and 0 to 3 days (Fig. 3).

### OPAS

Mean OPAS assessment scores for key questions are shown in Supplementary Figs. S1A and S1B. On day 2, SAF312-treated participants reported less intensity of eye pain (lower scores for when it was least painful and on average). No difference in OPAS scores was observed between SAF312 and vehicle when it was most painful (Supplementary Fig. S1A). Further, at an individual patient-level analysis of the eye pain on average at day 2, 53% (*n* = 21) of vehicle-treated patients reported a reduction in pain scores when treated with SAF312 after crossover (Supplementary Fig. S1B). As OPAS was an exploratory endpoint that has never been used in this patient population, the study was never powered for detection of any difference, and no statistical analysis was planned or performed in these data.

### Safety Assessment

All participants completed the study; there were no drug or study discontinuations due to AEs in the study, and no SAEs or deaths were reported. The determination of whether an AE was drug related or not was up to the investigators’ discretion. Moreover, it was determined based on whether the AE was consistent with those AEs typically seen and expected with the PRK procedure. A total of 10 patients (25%) experienced at least one treatment-emergent AE. Due to the crossover design, some patients accounted for the AEs in both SAF312 and vehicle treatment groups. Eight patients experienced AEs during treatment with SAF312, and five patients experienced AEs during treatment with vehicle. With SAF312 and vehicle treatments, 8 of 40 and 5 of 40 participants had at least one AE, respectively (Table 3). There were six ocular AEs, all of mild intensity, in four participants; none were suspected to be related to either SAF312 or vehicle. In total, five of the six ocular AEs were considered by the investigator to be related to the PRK procedure. There were 12 nonocular AEs reported in seven participants; the majority were mild; none were suspected to be drug related (Table 4).

**Table 3.** Overall Incidence of AEs

Characteristic	Vehicle (*N* = 40), *n* (%)	SAF312 2.5% (*N* = 40), *n* (%)	Total (*N* = 40), *n* (%)
Participants with at least one AE	5 (12.5)	8 (20.0)	10 (25.0)
Participants with at least one ocular AE	3 (7.5)	3 (7.5)	4 (10.0)
Participants with at least one nonocular AE	2 (5.0)	6 (15.0)	7 (17.5)
Number of AEs	5	12	18
Number of ocular AEs	3	3	6
Number of nonocular AEs	2	9	12
Number of mild severity AEs	4	8	13
Number of moderate-severity AEs	1	4	5
Number of severe AEs	0	0	0
Number of PRK-related AEs	3	2	5
Number of drug-related AEs	0	0	0

Nonocular AEs and mild severity AEs were reported in one participant prior to drug (after signing the informed consent and before administration of any study drug). An AE starting in one period and continuing into the next period was counted only in the onset period. Due to the crossover design, some patients accounted for the AEs in both SAF312 and vehicle treatment groups.

**Table 4.** Incidence of AE by Preferred Term

	Vehicle (*N* = 40)			SAF312 2.5% (*N* = 40)			No Drug (Prior to Drug)^a^ (*N* = 40)			
AEs by Preferred Term	*n* (%)	Severity	Procedure Related	*n* (%)	Severity	Procedure Related	*n* (%)	Severity	Procedure Related	Total (*N* = 40) *n* (%)
Participants with at least one AE	5 (12.5)			8 (20.0)			1 (2.5)			10 (25)
Ocular AEs										
Corneal infiltrate^b^	1 (2.5)	Mild	Yes	0			0			1 (2.5)
Corneal opacity^c^	1 (2.5)	Mild	Yes	1 (2.5)	Mild	Yes	0			1 (2.5)
Punctate keratitis^d^	1 (2.5)	Mild	Yes	1 (2.5)	Mild	Yes	0			1 (2.5)
Posterior vitreous detachment	0			1 (2.5)	Mild	No	0			1 (2.5)
Nonocular AEs										
Headache	1 (2.5)	Moderate	No	1 (2.5)	Mild	No	1 (2.5)	Mild	No	3 (7.5)
Arthralgia^e^	0			1 (2.5)	Mild	No	0			1 (2.5)
Nasopharyngitis^e^	0			2 (5.0)	Mild	No	0			2 (5.0)
Oropharyngeal pain^e^	0			1 (2.5)	Mild	No	0			1 (2.5)
Tinnitus	1 (2.5)	Mild	No	0			0			1 (2.5)
Sinus congestion	0			1 (2.5)	Moderate	No	0			1 (2.5)
Sinusitis	0			1 (2.5)	Moderate	No	0			1 (2.5)
Pyrexia^f^	0			1 (2.5)	Moderate	No	0			1 (2.5)
Vomiting^f^	0			1 (2.5)	Moderate	No	0			1 (2.5)

An AE starting in one period and continuing into the next was counted only in the onset period. One participant reported mild headache prior to drug. No deaths or serious AEs were reported. Of the 40 participants in this study, 8 and 5 had AEs while on SAF312 and vehicle, respectively. Due to the crossover design, some patients accounted for the AEs in both SAF312 and vehicle treatment groups.

a No drug, or baseline-emergent AE: AEs encountered after signaling the informed consent and before the administration of any study drug (SAF312 or vehicle).

b A small corneal infiltrate ≤0.5 mm in superior cornea near limbus on postoperative day 5 that resolved after 7 days was reported in one participant.

c Corneal opacity (haze) in both eyes at the end-of-study visit 36 days after last dose was reported in one participant.

d Punctate keratitis in both eyes was reported in one participant, both on postoperative day 8, that had resolved at the next visit (∼3 months postoperatively).

e One participant had tinnitus during vehicle treatment, had transient arthralgia on day 3 of SAF312 treatment, and developed a common cold with nasopharyngitis and oropharyngeal pain 13 days after the last dose of SAF312.

f One participant developed pyrexia and vomiting at the same time 36 days after the study medication, which was SAF312.

### Visual Acuity Assessment

There were no notable differences in best-corrected visual acuity (BCVA) and uncorrected visual acuity (UCVA) between SAF312- or vehicle-treated eyes. The mean (SD) BCVA and UCVA in both the treatment groups at baseline, day 2, day 3, and end of study (EOS) are summarized in Table 5.

**Table 5.** Summary of Visual Acuity over Time

	Vehicle (*N* = 40), Mean (SD)				SAF312 2.5% (*N* = 40), Mean (SD)			
Statistic	Baseline	Day 2	Day 3	EOS	Baseline	Day 2	Day 3	EOS
BCVA (ETDRS letters)	89.7 (2.54)	82.2 (7.53)	73.1 (10.04)	88.7 (3.42)	88.9 (3.04)	82.1 (6.18)	72.1 (10.43)	89.1 (3.37)
UCVA (ETDRS letters)	46.9 (19.51)	79.0 (11.37)	66.2 (13.34)	84.1 (7.61)	46.6 (19.63)	79.0 (5.66)	62.8 (11.05)	85.7 (5.78)

Safety analysis set. ETDRS, Early Treatment Diabetic Retinopathy Study.

### Ocular Hyperemia

**Figure 4.**
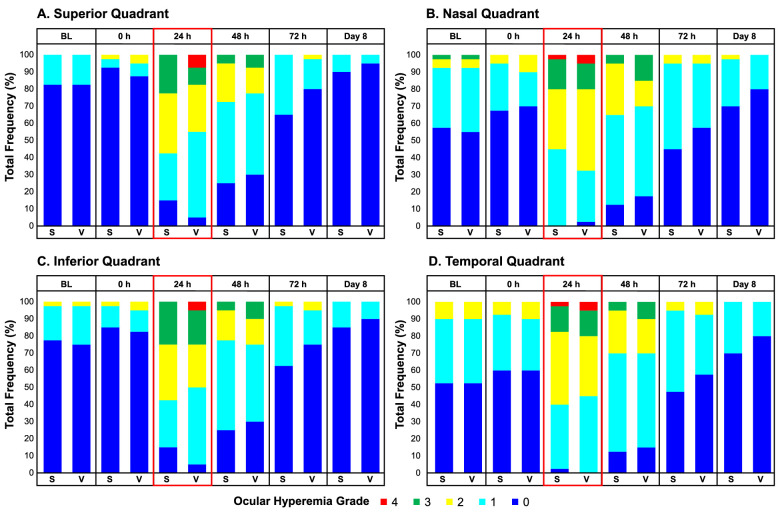
Less grade 4 hyperemia in SAF312- versus vehicle-treated eyes 24 hours postoperatively on day 2 in the superior quadrant with the most surgical manipulation (*P* = 0.040) and fewer grade 3 hyperemia in all quadrants at 48 hours. BL, baseline; S, SAF312; V, vehicle.

Twenty-four hours after PRK surgery (day 2 postoperatively), the frequency of grade 4 hyperemia in all quadrants was lower in SAF312-treated than vehicle-treated eyes (Fig. 4). The *P* value for this difference in the superior quadrant (with the most surgical manipulation) was 0.04 in favor of SAF312 over vehicle (Fig. 4A). Forty-eight hours after PRK surgery (day 3 postoperatively), SAF312-treated eyes had less grade 3 hyperemia than vehicle-treated eyes in all quadrants (Fig. 4).

### Size of Epithelial Defect by Slit-Lamp Exam (Wound Healing)

**Figure 5.**
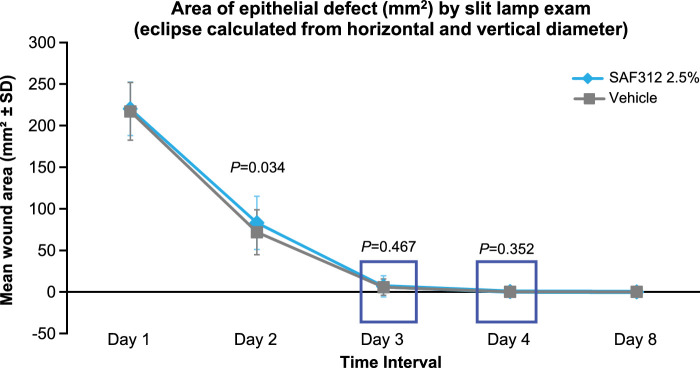
No delay in wound healing in SAF312-treated versus vehicle-treated eyes: wound healing was completed by 72 hours after PRK (day 4). *P* value compares the mean between both treatment groups, obtained from paired *t*-tests. Epithelial defect size was calculated by slit-lamp exam as the area of an ellipse calculated by maximum horizontal and vertical distance from center of epithelial defect.

There was no clinically significant difference between SAF312 and vehicle treatment toward the epithelial wound area at any time point (Fig. 5). At 24 hours after PRK (day 2), the difference between SAF312 and vehicle was 11.23 mm^2^ (83.02 vs. 71.79 mm^2^, *P* = 0.034). By 48 hours postoperatively (day 3), there was no difference between the SAF312- and vehicle-treated eyes, and the area of the wound was pinpoint. Almost all eyes had completely healed by 72 hours (day 4), and the duration of healing was consistent with the expected time anticipated for complete healing after PRK^33^ (Fig. 5).

### Blink Rate, Tear Production, and Corneal Staining

No relevant differences were observed in blink rate, tear production, or corneal staining between the treatment groups. There were no clinically significant changes in blink rate or tear production at EOS when compared with baseline. Most participants had normal corneal staining (grade 0) at baseline, day 8, and EOS, with no clinical difference observed between eyes treated with SAF312 and vehicle.

### Pharmacokinetic Results

The median T_max_ was at 0.459 hours and 0.467 hours after the 1st (day 1) and 13th (day 4) doses of SAF312 2.5%, respectively. Arithmetic mean C_max_ (percent coefficient of variation [CV%]) following the first dose on day 1 was 0.454 ng/mL (49.9%) and 2.40 ng/mL (63.5%; i.e., 5.3-fold higher) after the 13th dose on day 4. Corresponding mean AUC_last_ (CV%) values were 0.638 ng⋅h/mL (46.0%) and 4.38 ng⋅h/mL (67.0%), representing a 6.9-fold increase over the 4 days (Supplementary Table S4).

## Discussion

This phase 2 PoC study demonstrated that administration of topical SAF312 2.5% four times daily for 3 days was effective in reducing ocular pain in the immediate postoperative period after PRK surgery. Both study primary efficacy endpoints achieved statistical significance favoring SAF312 over vehicle, with a clinically meaningful reduction in mean VAS pain severity scores at the time points of (a) 6 hours after one drop of SAF312 at the end of surgery and (b) average VAS scores over 0 to 12 hours postoperatively after two eye drops (one immediately after surgery and one 6 hours later). These findings are clinically significant considering that acute pain after PRK begins shortly after the procedure and lasts until reepithelization is complete; intense pain is typically experienced within the first 12 hours of surgery, with the peak occurring around 4 to 6 hours after surgery.^32^^,^^34^

The efficacy of SAF312 versus vehicle as measured with VAS pain scores throughout 72 hours after the PRK was also assessed as the primary endpoints were set only for the first 12 hours. No significant differences in pain intensity between SAF312 and vehicle group were observed from 36 to 72 hours post-PRK. It is important to reduce both the maximum and overall pain that participants experience during the immediate postoperative period. The difference in VAS pain scores at 6 hours and averaging over 0 to 12 hours for SAF312-treated eyes was 11.1 (34.63 vs. 45.76) points and 8.56 (30.90 vs. 39.46) points lower than for vehicle-treated eyes, respectively. It has been reported that analgesic interventions following surgery that provide a change of 10 out of the 100-mm pain VAS score signifies a minimal clinically important difference, and a VAS ≤33 indicates acceptable pain control.^35^ It is noteworthy to highlight that a mean difference of >10 points in VAS score in favor of SAF312 over vehicle was noted at 1, 6, and 30 hours post-PRK; VAS pain scores with SAF312 treatment were maintained below 40 points for the entire 72-hour post-PRK period. In the current study, pain peaked with vehicle at 6 hours, with a VAS score of 45.76 points, and in the SAF312 group at 18.5 hours, with a VAS score of 39.12 points, indicating that SAF312 provided greater analgesic efficacy at the period of maximum pain intensity.

The reduction in pain provided by topical ocular SAF312 is comparable to previous reports for NSAID eye drops and oral opioids. In a study that evaluated diclofenac 0.1% and ketorolac 0.5% applied 2 hours before PRK and continued every 6 hours for 2 days, the VAS pain score (0–10 scale) at 24 hours post-PRK was 4.94 and 6.59 points, respectively.^36^ Preoperative administration of either one drop of topical diclofenac 0.1% or ketorolac 0.5% 30 minutes prior to PRK resulted in a 4.0-point (−57%) reduction compared with placebo (7.0 vs. 3.0 points for both groups) at day 1 on a 0 to 10 pain VAS scale.^9^ Similarly, with topical ketorolac 0.4% or oral naproxen sodium 220 mg every 12 hours for 72 hours following PRK, the mean pain scores (0–10 numerical rating scale [NRS]) were 2.02 and 2.07 points for the first 24 hours and 3.21 and 5.17 points for 24 to 48 hours post-PRK, respectively.^37^ Treatment with nepafenac 0.1%, ketorolac 0.4%, and bromfenac 0.09% eye drops three times daily after PRK resulted in a mean reduction of pain by 1.13, 0.31, and 0.42 compared with before NSAID drops on a 0 to 10 VAS scale at day 1.^38^ In a meta-analysis, pain relief from chronic low-back pain was reported to be better with tramadol compared to placebo with a mean 10.8-point difference on a 100-mm VAS.^39^

Consistent with the primary outcomes, participants with SAF312 treatment reported lower ORM use at all time points, and a higher proportion of participants on SAF312 had adequate pain control and did not require ORM during the first 24 hours postoperatively. SAF312, like topical NSAIDs, reduced the need for ORM and may be opioid sparing for optimal management of post-PRK pain.^34^^,^^40^

Topical ocular SAF312 2.5% treatment after PRK demonstrated a safety profile comparable with vehicle, with no serious or drug-related AEs and no delay in cornea reepithelization. There were no clinically relevant changes observed for visual acuity, blink rate, tear production, or corneal staining after SAF312 administration compared with vehicle. OPAS results also support the well-tolerated safety profile of SAF312, with participants reporting symptoms of redness, burning, sensitivity to light, and tearing to be slightly lower or comparable with vehicle treatment. Fewer SAF312-treated eyes showed severe conjunctival hyperemia on day 2. In contrast, ocular hyperemia and stinging are common AEs associated with topical ophthalmic NSAID treatment.^13^ Further, topical ophthalmic NSAIDs have the potential to delay corneal epithelial healing. When used for PRK pain management, topical agents such as diclofenac 0.1%,^40^^–^^42^ nepafenac 0.1/0.3%,^43^^,^^44^ ketorolac 0.4%/0.5%,^34^^,^^40^^,^^45^ and bromfenac 0.09%^38^ have been associated with delayed reepithelization.^4^ Previously, impaired healing of cornea incision injury in a TRPV1-deficient mouse has been reported.^46^ However, in a rabbit model of PRK,^47^ SAF312 did not exhibit delay in wound healing (manuscript under preparation). In agreement with the rabbit model of PRK, in this clinical trial, no delay in wound healing was observed between SAF312 and vehicle, and reepithelization was complete by 72 hours after PRK. This observation is clinically important, as the PRK procedure results in a controlled, large, epithelial defect up to 9 to 10 mm diameter and has a consistent healing time of 3 to 4 and up to 7 days. Thus, topical ocular SAF312 could be a potential, safe, and effective alternative to topical NSAIDs for optimized pain management.

TRPV1 channel antagonists, such as SAF312, offer advantages over other pain management strategies as they prevent ocular pain by blocking a receptor where pain is generated, as opposed to traditional analgesic drugs that either inhibit pain transmission (e.g., opiates) or suppress inflammation (e.g., NSAIDs). The study also confirmed that topical ocular administration of SAF312 2.5% led to a quick but very low level of systematic exposure, consistent with the findings of the FIH study.^18^

Owing to the low systemic exposure and the chemical properties of the SAF312 molecule, there seems to be a low risk on the CNS or other systemic side effects.

A different TRPV1 antagonist, AMG-517, promoted peripheral neuronal regeneration in rats after unilateral sciatic nerve injury by attenuation of TRPV1 activation. However, the role of SAF312 on the neuronal regeneration is yet to be explored.^48^

The strength of the study is its unique bilateral crossover PRK study design, where participants received either SAF312 2.5% or vehicle in the first treated eye and later switched study drug after PRK in the second eye, thereby acting as their own controls. Further, in this study, VAS and ORM use details were captured using a real-time electronic app, adding to its strength as written diaries are prone to error and fabrication. Thus, the results obtained using the ePRO device are more robust.^49^ Additionally, the pain reduction was observed with two different pain assessment tools (VAS and OPAS questionnaires), giving more confidence in the effect of SAF312 on post-PRK pain.

Study limitations include a relatively small sample size. In this study, pain after PRK was primarily assessed using electronic VAS on a 100-mm scale. Literature on a similar topic assessed pain after PRK using various methods besides VAS, including the NRS,^37^ Brief Pain Inventory,^50^ the McGill Pain Questionnaire,^50^^–^^52^ verbal rating scale,^53^ and the Wong–Baker FACES pain rating scale.^4^^,^^53^ A study also reported that a multidimensional questionnaire could provide improved analysis of pain after PRK.^54^ Use of different methods to assess pain limits direct comparison between these findings and other studies that evaluated use of topical agents for post-PRK pain.

In addition, participants in this study had the option of using ORM at any time during the study, which had the potential to confound the VAS pain severity efficacy results. To decouple the effect of ORM on VAS primary efficacy endpoints, any prespecified VAS reading taken within 4 hours of any ORM use was replaced with the VAS value recorded at the time the ORM was taken. Additional sensitivity analyses, including VAS scores after ORM use as missing and ignoring ORM use, were performed to examine the use of ORM on the study conclusions; all sensitivity analyses produced similar results and confirmed the robustness of the findings on drug effect (data not shown). Although bandage contact lens and corticosteroids are routinely used as postoperative standard of care (SOC), it serves as an additional confounding factor in the assessment of the effects of SAF312. This may have created some variability to the number of patients experiencing pain and the severity of their pain.

Thus, in this phase 2 clinical trial, topical ocular SAF312 2.5% (dosed four times daily for 3 days) showed efficacy in decreasing the severity of ocular pain associated with corneal epithelial defect after PRK surgery, no delay in wound healing, and a well-tolerated safety profile. The results of the study highlight that SAF312 can be a promising therapeutic option for patients with ocular pain after PRK. Further studies with additional patient-reported outcomes, such as quality of life, will provide better insights into the potential uses of topical SAF312 for ocular pain management.

## Supplementary Material

Supplement 1

Supplement 2
