# Supplementary material for: Topical Ocular TRPV1 Antagonist SAF312 (Libvatrep) for Postoperative Pain After Photorefractive Keratectomy
Source: Transl Vis Sci Technol. 2023 Mar 14;12(3):7. doi: 10.1167/tvst.12.3.7 (PMC10020951; doi:10.1167/tvst.12.3.7)
Supplement: Supplement 1 [file tvst-12-3-7_s001.pdf]

SUPPLEMENTARY DATA

Supplementary Appendix 1: Exclusion criteria

Supplementary Appendix 2: Other study assessments

Supplementary Appendix 3: Concomitant Medications/Significant non-drug therapies

Supplementary Table 1: Participant analyses set

Supplementary Table 2. VAS pain score over time

Supplementary Table 3. Amount of oral rescue medication use by body weight

Supplementary Figure 1: OPAS

Supplementary Table 4: PK data

Supplementary Appendix 1.

Exclusion criteria

Main exclusion criteria were as follows:

- Monocular participant (including amblyopia) or best-corrected visual acuity score worse than 20/80 (Snellen) or 55 ETDRS letters (Early Treatment Diabetic Retinopathy Study) at baseline
- Any systemic or ocular disease that affected wound healing (such as severe rheumatoid arthritis, diabetes or history of keloid formation) or history of ocular trauma, uveitis, infection, or inflammation in the 6 months prior to baseline. Especially for diabetes: participants with severe diabetes, uncontrolled diabetes, diabetic keratopathy, diabetic retinopathy, diabetic macular edema, diabetic nephropathy, diabetic foot ulcers or other systemic complications of diabetes were excluded. Participants with mild, well-controlled diabetes with no evidence of ocular or systemic complications of diabetes were included
- Participants with active inflammatory or infectious ocular conditions, severe or progressive retinal disease, and use of topical or systemic steroids, or use of coumadin or similar drugs within the last 6 months prior to baseline
- Participants with any corneal dystrophy (epithelial, stromal or endothelial) or any cornea disease (including significant scarring (at the discretion of the Investigator), ocular herpes or pterygium)
- Previous refractive or corneal surgery (such as LASIK, PRK, radial keratotomy, pterygium removal, corneal transplantation)
- History of allergic or hypersensitivity reaction or significant AEs to any of the drugs used in this study including tetracaine or similar topical ocular anesthetic, NSAIDs and aspirin, oral analgesic (including acetaminophen and codeine), antibiotics, steroids and inability to tolerate or wear bandage contact lens

- Concurrent therapy or history of chronic therapy or abuse of systemic or ocular NSAIDs, analgesics, pain medication (including gabapentin or pregabalin and similar), opiates or cannabis
- Participants who used any topical eye medication except for lubricating eye drops within two weeks prior to surgery in the study eye were excluded. Participants meeting any of the following were excluded:
    - Usage of topical NSAIDs during 30 days before baseline, OR
    - Systematic/chronic usage of systemic NSAIDS within 30 days prior to baseline, OR
    - Occasional usage of systemic NSAIDS within 3 days prior to baseline, OR
    - Usage of ocular cyclosporine (or similar medication) within the 3 months prior to surgery
- Participants with body weight <50 kg, or those whose body mass index (BMI) is not within the range of 18–35 kg/m^2

  BMI=body weight (kg)/ $[\text{height (m)}]^2$
- Pregnant or nursing (lactating) women. Women of child-bearing potential, defined as all women physiologically capable of becoming pregnant, unless they are using basic methods of contraception during dosing of investigational drug

No additional exclusions were applied by the Investigator, in order that the study population was representative of all eligible participants

Supplementary Appendix 2.

Other study assessments

ORM use was recorded in the eCRF and reconciled with participant entries in the provided electronic device. OPAS is a paper questionnaire consisting of 27 questions designed to measure ocular pain intensity in participants with eye pain. The rating scale of the overall pain severity ranged from 0 (no pain) to 10 (severe pain) or for frequency of symptoms from 0% (never) to 100% (all the time).[47] Participants completed the questionnaire on Days 2, 4, and 8. BCVA and (UCVA were measured at each visit using an ETDRS VA chart at 4 or 1 m (for participants who cannot read the 4 m chart). Ocular hyperemia was assessed in 4 regions (superior, inferior, temporal, nasal) of each eye using the slit lamp exam and graded according to the McMonnies redness scale (0-5 photographic scale, with higher scores indicating greater degree of conjunctival redness). Size of epithelial defect was assessed by slit lamp exam with the elliptical area of epithelial wound size was calculated as follow: area (mm^2) = width×height×π, from the width and height of the epithelial defect measured at the slit lamp. Blink rate, tear production (Schirmer's test without anesthesia), and corneal staining tests were performed in study centers according to the protocol. The Schirmer's test was performed without anesthetic[1] in both eyes simultaneously. The test was performed for 5 minutes with the eyes closed and tear secretion was measured in mm by the length of strip wetted by tears. Corneal staining was performed by gently touching the wet end of an impregnated sodium fluorescein strip to the inferior conjunctival sac. The patient blinked several times to ensure dispersion of the dye throughout the tear film prior to grading on a scale of 0–3 (0 = Normal, No staining; 1 = Mild, Superficial stippling micropunctate staining; 2 = Moderate, Macropunctate staining with some coalescent areas and 3 = Severe, Numerous coalescent macropunctate areas and/or patches) for each of five zones (central plus four quadrants).[2] Blood samples for PK analysis were collected at the timepoints defined in the visit schedule and PK parameters (C_{max}, T_{max}, AUC_{last}) were determined per-protocol using the actual recorded sampling times and non-

compartmental method with Phoenix™ WinNonlin (version 6.4). SAF312 was quantified in plasma using a validated liquid chromatography-mass spectrometry/mass spectrometry method; the lower limit of quantification was 0.05 ng/mL.

Supplementary Appendix 3.

Concomitant Medications/Significant non-drug therapies:

A list of all medications used by the patients post PRK:

Study procedures:

- Topical drops:
  - Acetaminophen/Codeine (rescue medication)
  - Artificial Tears
  - Prednisolone
- Anesthetic:
  - Tetracaine (used immediately before the PRK procedure and before IOP measurement at baseline, 8-day post PRK)
- Antibiotics
  - Moxifloxacin (Ofloxacin or Polytrim alternative)
- Temporary/permanent punctal plugs

Others:

- Avenova Lid Scrub and Hypochlorous acid (mild meibomian gland dysfunction)
- Fluticasone Propionate (sinus congestion)
- Phentermine hydrochloride (weight loss)
- Drospirenone and Ethinylestradiol (birth control)

Supplementary Table 1. Participant analysis sets

	Vehicle/ SAF312 2.5% N=20	SAF312 2.5%/ Vehicle N=20	Total N=40
**Participants randomized, n (%)**	20 (100)	20 (100)	40 (100)
**Safety analysis set, n (%)**	20 (100)	20 (100)	40 (100)
**PK analysis set, n (%)**	19 (95)	18 (90)	37 (93)
**Primary analysis set, n (%)**	15 (75)	15 (75)	30 (75)
**Secondary analysis set, n (%)**	20 (100)	20 (100)	40 (100)

Safety analysis set: All participants who received study drug and with no protocol deviations with relevant impact on safety. Primary analysis set, n (%): All participants who experienced no protocol deviations with relevant impact on VAS data. Secondary analysis set, n (%): All participants who experienced no protocol deviations with relevant impact on secondary data. The first 10 randomized participants were excluded from the primary analysis (5 participants from each treatment sequences), resulting in an analysis set of 30 (75%) participants randomized in 1:1 ratio to both treatment sequences. These 10 participants were excluded from the primary analysis as they did not have evaluable primary endpoint data due to an ePRO (electronic participant reported outcome) device failure. As incorrect attribution of pain measurements has a direct effect on data interpretation, this led to switching to a different ePRO device which was used successfully for the primary and secondary endpoint collection in the next 30 participants.

PK analysis set, n (%): All participants with at least one available valid (i.e., not flagged for exclusion) PK concentration measurement, who received SAF312 and experienced no protocol deviations with relevant impact on PK data. The PK analysis set consisted of 37 participants, resulting from exclusion of 3 IMP non-compliant participants (1 Vehicle/SAF, 2 SAF/Vehicle).

Supplementary Table 2. Visual analog scale pain score over time

Hours (h) Postoperatively	Mean* (SE) of VAS pain score		Mean (SE) of difference	P-value
	SAF312 2.5%	Vehicle		
**1.0h**	n=27<br>12.89 (4.16)	n=22<br>27.32 (4.43)	−14.4 (4.32)	**0.001**
**3.0h**	n=28<br>34.94 (4.13)	n=28<br>43.73 (4.14)	−8.79 (3.97)	**0.028**
**6.0h**	n=30<br>34.63 (4.05)	n=29<br>45.76 (4.10)	−11.1 (3.88)	**0.005**
**6.5h**	n=30<br>31.80 (4.05)	n=27<br>38.44 (4.17)	−6.64 (3.95)	**0.095**
**9.0h**	n=30<br>30.70 (4.05)	n=29<br>36.62 (4.10)	−5.92 (3.88)	0.129
**12.0h**	n=26<br>29.50 (4.20)	n=29<br>37.30 (4.10)	−7.80 (4.04)	**0.054**
**18.0h**	n=27<br>34.41 (4.16)	n=25<br>41.44 (4.26)	−7.02 (4.11)	**0.089**
**18.5h**	n=28<br>39.12 (4.12)	n=29<br>44.20 (4.10)	−5.07 (3.95)	0.201
**24.0h**	n=30<br>25.03 (4.05)	n=29<br>34.23 (4.10)	−9.19 (3.88)	**0.019**
**24.5h**	n=29<br>27.92 (4.09)	n=29<br>33.30 (4.10)	−5.37 (3.92)	0.172
**30.0h**	n=29<br>26.27 (4.09)	n=28<br>41.96 (4.14)	−15.7 (3.96)	**0.000**
**36.0 h**	n=29<br>35.67 (4.09)	n=28<br>37.25 (4.13)	−1.58 (3.95)	0.689
**42.0h**	n=28<br>21.68 (4.12)	n=28<br>20.32 (4.13)	1.36 (3.98)	0.734
**48.0h**	n=28<br>15.05 (4.12)	n=29<br>17.99 (4.10)	−2.94 (3.96)	0.458
**54.0h**	n=29<br>12.38 (4.09)	n=29<br>13.15 (4.10)	−0.77 (3.92)	0.844

**60.0h**	n=30	n=28		
	11.80 (4.05)	11.19 (4.14)	0.61 (3.92)	0.876
**66.0h**	n=29	n=27		
	8.58 (4.09)	8.78 (4.18)	−0.20 (4.00)	0.959
**72.0h**	n=30	n=29		
	5.13 (4.05)	3.58 (4.10)	1.56 (3.88)	0.689

Primary analysis set.

VAS pain was analyzed using a longitudinal mixed model repeated measures (MMRM) that accounted for the crossover effect with repeated measurement for VAS, with all assessments post-op for each period included in the analysis up to 3 days post-op. The model included fixed effects of treatment, hours post-op, period, sequence, the treatment-by-hour post-op interaction, the hours post-op-by-period for each participant were accounted for using random effects for participants, for participant-treatment combinations within a sequence, and for participant-timepoint combinations within a sequence. Any recorded VAS pain score at timepoints within 4 h after the use of rescue medication were imputed by the record taken prior to the use of rescue medication (LOCF).

*Least square arithmetic mean.

LOCF, last observation carried forward; n, number of participants at each timepoint; post-op, postoperative; SD, standard deviation; SE, standard error; VAS, visual analog scale.

Supplementary Table 3. Amount of oral rescue medication use (mg/kg body weight)

**ORM**<br>mg/kg body weight	**SAF312 2.5%**<br>**N=40**	**Vehicle**<br>**N=40**	**%**<br>**Change**	***P* value**
**0–6h post-op**				
Mean (SD)	3.32 (4.684)	4.41 (5.038)	–25%	0.09*
Range	0.0–19.2	0.0–17.1		
**0–12h post-op**				
Mean (SD)	6.10 (8.062)	7.39 (8.478)	–17%	0.21
Range	0.0–28.7	0.0–28.7		
**0–24h post-op**				
Mean (SD)	10.17 (12.335)	12.41 (14.130)	–18%	0.05*
Range	0.0–47.9	0.0–57.5		
**0–48h post-op**				
Mean (SD)	17.59 (19.850)	20.51 (23.991)	–14%	0.05*
Range	0.0–76.6	0.0–95.8		
**0–72h post-op**				
Mean (SD)	18.70 (21.509)	22.06 (25.527)	–15%	0.06*
Range	0.0–87.4	0.0–105.4		

Secondary analysis set
% Change is calculated by ORM use of (SAF−Vehicle)/Vehicle.
P values compare the mean between both treatment groups, obtained from Wilcoxon signed rank test.
Each acetaminophen/codeine tablet is considered as 330 mg in the calculation of amount (mg/kg of body weight)
ORM, oral rescue medication; post-op, postoperative; SD, standard deviation.

Supplementary Table 4. Summary statistics of SAF312 PK parameters after topical ocular administration of SAF312 2.5%

Profile Day	Statistic	C_{max} (ng/mL)	T_{max} (h)	AUC_{last} (ng*h/mL)[1]
	N	34	34	34
	Mean (SD)	0.454 (0.227)	-	0.638 (0.293)
**1**	CV%	49.9	-	46.0
	Median	0.375	0.459	0.569
	Min, Max	0.195, 1.05	0.167, 2.00	0.261, 1.58
	N	33	33	31
	Mean (SD)	2.40 (1.53)	-	4.38 (2.94)
**4**	CV%	63.5	-	67.0
	Median	2.07	0.467	3.42
	Min, Max	0.639, 7.56	0.00, 2.08	1.18, 14.5

PK analysis set
[1]Median T_{last} (range): Day 1, 2 h (1.95–2.05 h); Day 4, 2.00 h (1.77–2.10 h)
C_{last} not shown
AUC, area under the curve; C_{max}, maximum plasma concentration; CV, coefficient of variation; PK, pharmacokinetic; SD, standard deviation.

Reference:

1. Karampatakis V, Karamitsos A, Skriapa A, Pastiadis G. Comparison between normal values of 2- and 5-minute Schirmer test without anesthesia. *Cornea.* 2010;29(5):497-501.

2. American Academy of Ophthalmology. National Eye Institute (NEI) grading scale. Available at NEI/Industry Grading System - American Academy of Ophthalmology (aao.org). Accessed on 15 Dec 2022.
