# Supplementary material for: Topical Ocular TRPV1 Antagonist SAF312 (Libvatrep) for Postoperative Pain After Photorefractive Keratectomy
Source: Transl Vis Sci Technol. 2023 Mar 14;12(3):7. doi: 10.1167/tvst.12.3.7 (PMC10020951; doi:10.1167/tvst.12.3.7)
Supplement: Supplement 2 [file tvst-12-3-7_s002.pdf]

Supplementary Figure 1. A) Key OPAS assessments; B) Patient-level analysis – OPAS score for eye pain on average at Day 2

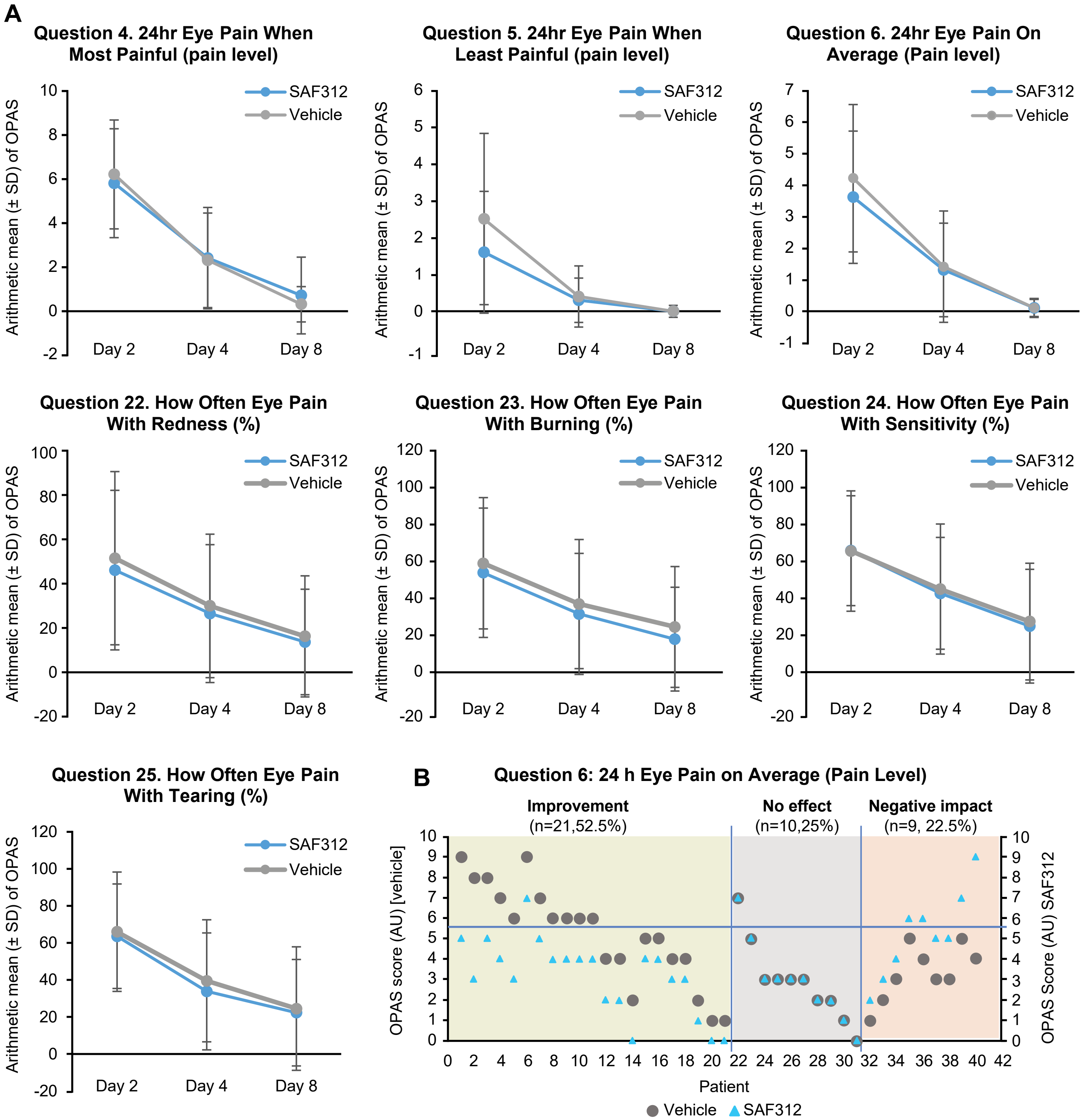

Secondary analysis set. A) Arithmetic mean (±SD) of OPAS over time. The OPAS is a questionnaire with a rating scale of the overall pain severity rated from 0 (no pain) to 10 (severe pain) or for frequency of symptoms from 0% (never) to 100% (all the time) as per the survey. B) Approximately 53% (n=21) Vehicle-treated patients reported reduction in pain scores when treated with SAF312 (n=21, 52.5%); 25% patients (n=10), mostly comprising of those patients with moderate to mild pain levels (OPAS scores 5-2 AU) did not report any difference (experienced similar pain levels) when switched to SAF312 use, while 22.5% patients (n=9) reported slightly enhanced pain levels with SAF312 compared to Vehicle
AU, arbitrary units; H, hour; n, number of patients; OPAS, ocular pain assessment survey; SD, standard deviation.
